# Association of *STAT4* Gene Polymorphisms (rs10181656, rs7574865, rs7601754, rs10168266) and Serum STAT4 Levels in Age-Related Macular Degeneration

**DOI:** 10.3390/biomedicines12010018

**Published:** 2023-12-20

**Authors:** Tomas Blekeris, Greta Gedvilaite, Kriste Kaikaryte, Loresa Kriauciuniene, Dalia Zaliuniene, Rasa Liutkevciene

**Affiliations:** 1 Medical Faculty, Medical Academy, Lithuanian University of Health Sciences, LT-50161 Kaunas, Lithuania; tomas.blekeris@stud.lsmu.lt (T.B.); kriste.kaikaryte@lsmuni.lt (K.K.); 2 Neuroscience Institute, Medical Academy, Lithuanian University of Health Sciences, LT-50161 Kaunas, Lithuaniarasa.liutkeviciene@lsmuni.lt (R.L.); 3 Ophthalmology Department, Medical Academy, Lithuanian University of Health Sciences, LT-50161 Kaunas, Lithuania; dalia.zaliuniene@lsmuni.lt

**Keywords:** age-related macular degeneration, *STAT4* gene polymorphisms

## Abstract

Age-related macular degeneration (AMD) is a progressive degenerative disease that affects the central part of the retina: the macula. AMD is the most common cause of central vision loss in industrialized countries. Increasing attention is being paid to the study of genetic factors that may influence the manifestation of AMD. STAT4 protein is involved in the pathogenesis of numerous inflammatory processes, so we decided to investigate the association between *STAT4* gene polymorphisms (rs10181656, rs7574865, rs7601754, and rs10168266) and age-related macular degeneration. Purpose: To investigate the association between *STAT4* (rs10181656, rs7574865, rs7601754, and rs10168266) gene polymorphisms and STAT4 serum levels in patients with age-related macular degeneration. Methods and participants: The study included 150 individuals with early AMD, 150 individuals with exudative AMD, and 200 healthy subjects. DNA was extracted from peripheral blood leukocytes using the DNA salting-out method, and the genotyping was performed using a real-time polymerase chain reaction (RT-PCR) method. STAT4 serum levels were evaluated using the ELISA method. Statistical analysis was performed using “IBM SPSS “Statistics 29.0” software”. Results: The study revealed no statistically significant differences in the distribution of genotypes and alleles for the *STAT4* polymorphisms (rs10181656, rs7574865, rs7601754, and rs10168266) between patients with AMD and the control group. Similarly, a gender-based analysis did not yield any significant differences in the genotype or allele frequencies. Age group comparisons also showed no statistically significant variations in the presence of these *STAT4* polymorphisms between AMD patients and the control group. However, notably, individuals with exudative AMD displayed lower levels of serum STAT4 in comparison to the control group (median (IQR): 0.118 (0.042) vs. 0.262 (0.385), *p* = 0.005). Conclusion: Investigating *STAT4* gene polymorphisms (rs10181656, rs7574865, rs7601754, and rs10168266) did not reveal a significant association with AMD. However, further analysis demonstrated intriguing findings regarding serum STAT4 levels. Exudative AMD patients with at least one G allele of the *STAT4* rs10181656 exhibited significantly lower serum STAT4 levels than the control group subjects (*p* = 0.011). Similarly, those with at least one T allele of STAT4 rs10168266 had lower serum STAT4 levels compared to the control group subjects (*p* = 0.039). These results suggest a potential link between specific *STAT4* genotypes and serum STAT4 levels in exudative AMD patients, shedding light on a novel aspect of the disease.

## 1. Introduction

Age-related macular degeneration (AMD) is a progressive degenerative disease affecting the central part of the retina: the macula. AMD is the most common cause of central vision loss in developed countries. AMD usually occurs in people older than 55 years. As the population ages, AMD is becoming an increasingly important and sensitive disease worldwide [1]. It is estimated that the number of people with AMD will increase from 196 million in 2020 to 288 million in 2040 [2]. The effects of various factors influence the occurrence of AMD. Age has the greatest influence on the development of the disease, but comorbidities, lifestyle, smoking, hypertension, cholesterol, and high BMI are also important [3,4,5]. Currently, there is no cure for AMD, but it is possible to effectively halt the progression of the disease, and for this early diagnosis of the disease is important. Currently, there is no treatment for dry AMD. Vascular endothelial growth factor inhibitors are the main treatment for exudative AMD [2]. To prevent the onset and progression of the disease, control of modifiable risk factors is important. The pathogenic mechanisms of AMD are not well understood, but it is well established that the development of AMD is influenced by lifestyle, environment, metabolism, and genetic factors [6]. The risk factors for AMD are very similar to the risk factors for cardiovascular disease: age, smoking, high cholesterol, hypertension, and high body mass index [3,4,5]. The drusen that occur in AMD are composed of the same protein complexes as atherosclerotic plaques, so it is only natural that research be conducted to determine whether cardiovascular disease is associated with the manifestation of AMD [4]. The European Ocular Epidemiology (E3) Consortium conducted the European Eye-Risk Project, which found that elevated high-density lipoprotein cholesterol was associated with AMD risk and larger drusen area. In contrast, higher triglycerides were associated with smaller drusen and lower AMD risk. Diabetes mellitus is also being investigated as a potential risk factor for AMD, as both conditions are associated with increased oxidative stress [3]. The increasing use of smart devices that emit blue light in everyday life has been reported in the literature as a potential risk factor for eye disease [2].

Currently, increasing attention is being paid to the study of genetic factors that may influence the manifestation of AMD. The main pathogenetic mechanisms leading to the development of AMD are the formation of drusen, local inflammation and neovascularization. Stat4, a transcription factor known for its regulatory role in pro-inflammatory signaling, promotes great vessels (GV) vasculogenesis in zebrafish. Some of the Stat4-related pro-inflammatory factors may be involved in large vessel vasculogenesis. Recent studies have revealed a paradigm in which the endogenous mechanisms of pro-inflammatory factors contribute to the maintenance of normal tissue homeostasis [7]. Therefore, pro-inflammatory cytokines and chemokines have also been reported to be expressed in the hearts of infants with congenital heart disease and large vascular defects [8]. But STAT4 is expressed at low levels in cultured human umbilical vein endothelial cells and is tyrosine phosphorylated by interferon [9]. We therefore hypothesized that STA4 may play an important role in the pathogenesis of AMD by influencing the formation of new vessels and wanted to test whether low or high serum STAT4 levels influence the development of AMD.

STAT4 protein is also involved in the pathogenesis of many inflammatory and autoimmune diseases and has been associated with rheumatoid arthritis and systemic lupus erythematosus [10]. Signal Transducers and Activators of Transcription (STAT) are a family of proteins responsible for regulating numerous processes related to cell proliferation, differentiation, apoptosis, and immune response. STAT proteins reside in the cytoplasm and are activated by cytokines and growth factors. After activation, proteins translocate to the nucleus, bind to specific promoters, and regulate gene transcription. The STAT protein family consists of seven members, STAT1, STAT2, STAT3, STAT4, STAT5A, STAT5B, and STAT6, which have been identified in human and mouse genomes. The size of these proteins varies between 750 and 850 amino acids [11].

Each protein of the STAT family plays a different role in signal transduction and is crucial for the cellular response to various cytokines [12]. The *STAT4* gene is located on the long arm of chromosome 2 at position 2q32.2–2q32.3. STAT proteins consist of different regions that differ in structure and function. The N-terminal domain is 124–145 amino acids long. N-terminal and SH2 dimers are mediators of dimerization, allowing the free ends to form STAT dimer complexes and bind to DNA [11]. The SH2 domain is a key mediator of dimerization and a critical factor at the STAT protein–receptor interface. The coiled-coil domain consists of four long ⍺-helices and interacts with other proteins and STAT protein domains [12]. The DNA-binding domain is the C-terminal portion of the protein [13]. The transcription activation domain increases transcriptional activity after serine phosphorylation. The linker domain connects the DNA-binding domain to the SH2 domain [11].

The fourth member of the STAT protein family, the transcriptional signal transducer and activator STAT4, is localized in the cytoplasm. Various cytokines phosphorylate STAT4 after membrane binding and dimerized STAT4 migrates to the nucleus to regulate gene expression [14]. STAT4 is involved in developing many autoimmune and inflammatory diseases and plays a key role in tumor and inflammatory processes. STAT4 protein is crucial for targeting interleukin-12; therefore, IL-12 activates STAT4. The major functions of IL-12 are the production of interferon-ɣ (IFN-ɣ) and the differentiation of Th1 cells into Th17 [15]. Several single nucleotide polymorphisms of the *STAT4* gene have been associated with diseases such as rheumatoid arthritis, Sjogren’s, asthma, and systemic lupus erythematosus [10]. In addition to the already known associations of the *STAT4* gene with the aforementioned diseases, it is important to find other diseases whose development may be influenced by the *STAT4* gene. As a result, new studies are being conducted with the *STAT4* gene. In one such study, conducted among the Chinese Han population, it was reported that the *STAT4* gene polymorphisms rs3821236, rs11893432, rs11889341, rs7574865, and rs897200 are associated with the risk of developing type 2 diabetes [16]. *STAT4* gene expression is also associated with the risk of type 1 diabetes. A study conducted in Poland found an association between the rs7574865 polymorphism of the *STAT4* gene and the risk of type 1 diabetes in a population of Polish European children [17]. The study included 1438 individuals whose genotypes were compared, including 656 children with type 1 diabetes and 782 healthy adults as a control group. According to scientific research, *STAT4* gene expression is associated with the risk of both type 1 and type 2 diabetes [16,17]. In a study conducted in western China involving 725 individuals, *STAT4* polymorphisms rs7574865, rs10181656, rs10168266, and rs13426947 were also found to be associated with the risk of neuromyelitis optica spectrum disorder [18]. Our study uniquely integrated the analysis of single nucleotide polymorphisms (SNPs) with serum STAT4 levels in blood serum, providing a comprehensive approach to understanding AMD. This dual analysis considers both genetic predispositions, particularly in genes like *STAT4*, and systemic factors reflected in serum biomarkers. By exploring this interplay, our research aims to contribute novel insights into AMD pathogenesis, potentially informing diagnostic and therapeutic strategies. To discover new genetic markers associated with the development of AMD, we decided to investigate the single nucleotide polymorphisms rs10181656, rs7574865, rs7601754, and rs10168266 of the *STAT4* gene and determine their influence on the manifestation of AMD.

## 2. Methods

All subjects signed an agreement in accordance with the Declaration of Helsinki. The study was conducted in the Laboratory of Ophthalmology of the Institute of Neurosciences of the Lithuanian University of Health Sciences. The Kaunas Regional Biomedical Research Ethics Committee approved the study (approval numbers: 9 July 2015 No. BE-2-26 and 26 January 2017 No. P1-BE-2-26/2015).

A total of 500 subjects were studied, and two study groups were formed: a control group (*n* = 200) and a group of patients with AMD (n = 300). The patient group was divided into two subgroups: patients with early AMD (*n* = 150) and patients with exudative AMD (*n* = 150).

The control group consisted of individuals who had no ocular pathology at examination and agreed to participate in the study. The exclusion criteria for patients in the study were described in our previous study.

The exclusion criteria for patients with AMD were (1) related ocular diseases (high refractive error, cloudy cornea, or lens opacity (nuclear, cortical, and posterior subcapsular cataract), excluding minor opacities, and patients with intraocular lenses, keratitis, acute or chronic uveitis, glaucoma, late age-related macular degeneration, optic nerve disease); (2) systemic diseases (diabetes mellitus, oncological diseases, systemic tissue disorders, chronic infectious diseases, and conditions after organ or tissue transplantation); (3) color fundus photography because of the opacity of the optical system of the eye or because of the quality of fundus photography; (4) congenital color vision disorders were excluded by history; and (5) patients with epilepsy and taking sedatives.

The inclusion criteria for healthy patients were as follows: (1) no ophthalmic eye diseases detected in the detailed ophthalmic examination; (2) informed consent to participate. The exclusion criteria for healthy patients were as follows: (1) any ophthalmic diseases; (2) patients with epilepsy and taking sedatives.

Ophthalmic examination of all subjects in our study was performed as follows. Visual acuity (VA) was estimated from letter charts and reported in decimal notation. All patients were examined with slit-lamp biomicroscopy. Biomicroscopy was used to assess corneal and lens transparency. Intraocular pressure was measured at each examination. The patients were dilated with 1% tropicamide. After pupil dilation, funduscopy was performed with a direct monocular ophthalmoscope and slit lamp using a double aspheric lens of +78 diopters. The examination results were recorded on standardized forms developed for this study. Color fundus photographs were taken with a fundus camera at half wide angle (OPTON SBG, 30 degrees). Photographs were taken with the focus on the center of the fovea.

Optical coherence tomography was performed in all AMD patients (OCT), and fluorescein angiography was performed in patients with suspected late AMD after examination of the OCT. For this study, we used the classification system for AMD formulated in the previous Age-Related Eye Disease Study [6,19]: Early AMD consisted of multiple small drusen and multiple intermediate (63–124 μm diameter) drusen or abnormalities of the retinal pigment epithelium. Extensive intermediate drusen characterized early intermediate AMD and at least one large druse (≥125 μm diameter) or geographic atrophy that did not involve the center of the fovea. Exudative AMD was identified by the occurrence of geographic atrophy involving the fovea and/or any of the neovascular AMD features.

The control group consisted of individuals who had no ocular pathology at examination and agreed to participate in the study.

### 2.1. DNA Extraction and Genotyping

The DNA extraction and analysis of the *STAT4* gene polymorphisms were performed at the Ophthalmology Laboratory of the Neuroscience Institute of the Lithuanian College of Health Sciences. Blood samples were typically collected before 10 am. After obtaining the blood, the sample was immediately delivered to the laboratory. The sample was coded and labeled according to the laboratory’s instructions. Then, it was either used for DNA extraction or refrigerated for future use. For serum preparation, upon collection of the whole blood, it was left undisturbed at room temperature for 15–30 min to allow for clotting. Subsequently, the clot was removed by centrifuging the blood at 1000–2000× *g* for 10 min in a refrigerated centrifuge, resulting in the designated serum (i.e., supernatant). This serum was immediately transferred into a clean microcentrifuge tube using a pipette. Throughout handling, the samples were maintained at 2–8 °C. If the serum was not analyzed immediately, it was stored and transported at −20 °C or lower for further investigations. Approximately 3 mL of blood is required to extract approximately 250 μg of DNA, a quantity optimal for various tests, including TL-PCR. The DNA extraction was performed on the venous blood samples using the salting-out method. Briefly, venous blood samples (i.e., white blood cells) were collected and suspended in a buffer solution, followed by the addition of detergents to degrade cell membranes, proteinase K to hydrolyze proteins, and chloroform to deproteinize them. The DNA was then precipitated with ethanol. Additionally, the DNA concentration was determined using spectrophotometry. The 260/280, 260/230, and 260/325 absorbance ratios were used in assessing the DNA purity and identifying contaminants in the biological samples during DNA extraction. To ensure optimal accuracy, the readings ideally fell within the range of 0.1 to 1.0.

Single nucleotide polymorphisms (SNPs) were determined using TaqMan^®^ genotyping assays (Thermo Scientific, Pleasanton, CA, USA, Canada) and following the manufacturer’s. Genotyping of *STAT4* rs10181656, rs7574865, rs7601754, and rs10168266 was performed using real-time PCR (RT-PCR) according to the manufacturers, using a Step One Plus RT-PCR system (Applied Biosystems, Foster City, CA, USA) and an allele discrimination program. Into each of the 96 wells on the plate, we added 1.5 μL of the DNA samples and 8.5 μL of the PCR reaction mixture, along with the negative control. The program analyzed each genotype based on the fluorescence intensity of the different detectors (VIC and FAM).

### 2.2. ELISA

STAT4 levels were determined using an enzyme-linked immunosorbent assay (ELISA) with the Abbexa Signal Transducer And Activator Of Transcription 4 (STAT4) ELISA kit (UK, Cambridge).

In this method, a 96-well plate was precoated with an antibody. Following the addition of standards, test samples, and a biotin-conjugated reagent, the plate was incubated. Subsequently, an HRP-conjugated reagent was introduced, and the plate underwent another incubation. Wash buffer was used to remove any unbound conjugates at each stage. The HRP enzymatic reaction was quantified using a TMB substrate, resulting in a blue-colored product in the wells containing adequate STAT4, which transformed to yellow upon the addition of an acidic stop solution. The intensity of the yellow color was directly proportional to the amount of STAT4 bound to the plate. The optical density (OD) was measured at 450 nm using a microplate reader, enabling the calculation of the concentration of STAT4. The absorbance was measured at the required 450 nm, and the concentration was calculated from a calibration curve based on the standard solutions used.

### 2.3. Statistical Analysis

Statistical analysis of the data was performed using IBM SPSS Statistics 27.0. Data are presented as absolute numbers (percentages) and the median (IQR). The Mann–Whitney U test was used to detect differences between two independent groups. To compare the homogeneity of the genotype distribution of polymorphisms between AMD patients and controls, χ2 and Fisher’s and two-way criteria were used. Binary logistic regression analysis was used to estimate the odds ratio (OR) of AMD occurrence as a function of genetic inheritance patterns. The genetic models (codominant: heterozygotes vs. wild-type homozygotes and homozygotes vs. wild-type homozygotes; dominant: homozygotes with a rarer allele and heterozygotes vs. wild-type homozygotes; recessive: homozygotes with a rarer allele vs. wild-type homozygotes; recessive: homozygotes with a rarer allele vs. wild-type homozygotes) were included in the analysis. Homozygotes with rarer allele vs wild-type homozygotes and heterozygotes; supradominant: heterozygotes vs wild-type homozygotes vs homozygotes with rarer allele; an additive model was used to model the effect of each rarer allele on the development of AMD. This analysis was performed with a 95% confidence interval (CI) for the group with AMD. The Akaike information criterion (AIC) was evaluated to select the best inheritance model, with the lowest value indicating the best-fitting model. After Bonferroni correction, differences were considered statistically significant when *p* < 0.05/4 (*p* < 0.0125).

## 3. Results

This case–control study included 500 participants: 150 subjects in the early AMD group (average age: 71.49 years) and 150 subjects in the exudative AMD group (average age: 71.46 years). The control group comprised 200 healthy subjects (average age: 71.42 years) (Table 1).

### 3.1. Frequencies of STAT4 (rs10181656, rs7574865, rs7601754, and rs10168266) Genotypes and Alleles in Patients with AMD and Control Group

No statistically significant differences were found between the frequencies of *STAT4* (rs10181656, rs7574865, rs7601754, and rs10168266) genotypes and alleles in early AMD and control groups (Table 2).

Moreover, no statistically significant differences were found between the frequencies of the *STAT4* (rs10181656, rs7574865, rs7601754, and rs10168266) genotypes and alleles in the exudative AMD and control groups (Table 3).

### 3.2. STAT4 (rs10181656, rs7574865, rs7601754, and rs10168266) Genotypes and Allele Associations with Early and Exudative AMD

We analyzed *STAT4* (rs10181656, rs7574865, rs7601754, and rs10168266) genotypes and allele associations with early and exudative AMD. No statistically significant associations were found (Table 4 and Table 5).

### 3.3. STAT4 (rs10181656, rs7574865, rs7601754, and rs10168266) Genotypes and Allele Associations with Early and Exudative AMD by Gender

The allele frequency analysis showed no statistically significant differences between *STAT4* (rs10181656, rs7574865, rs7601754, and rs10168266) genotypes and alleles in the early AMD, exudative AMD, and control groups depending on gender (Table 6 and Table 7).

Binary logistic regression revealed that the association between *STAT4* (rs10181656, rs7574865, rs7601754, and rs10168266) in the early AMD and control groups by gender was not statistically significant (Table 8). Also, no statistically significant association was found between *STAT4* (rs10181656, rs7574865, rs7601754, and rs10168266) in the exudative AMD and control groups, depending on gender (Table 9).

### 3.4. Frequencies of STAT4 (rs10181656, rs7574865, rs7601754, and rs10168266) Genotypes and Alleles in Patients with AMD and in the Control Group by Age

Since AMD is a major cause of central vision loss in the developed world affecting 10% of people older than 65 years and more than 25% of people older than 75 years, we decided to divide our subjects into three groups, depending on age (Table 10). Our results showed that there were no statistically significant differences in *STAT4* (rs10181656, rs7574865, rs7601754, and rs10168266) genotypes and alleles among the early AMD, exudative AMD, and control groups, depending on age (Table 10).

### 3.5. STAT4 (rs10181656, rs7574865, rs7601754 and rs10168266) Genotype and Allele Associations with Early and Exudative AMD by Age

No statistically significant association was found between *STAT4* (rs10181656, rs7574865, rs7601754 and rs10168266) in early AMD, exudative AMD and control groups in ≤65-year-old subjects (Table 11).

Furthermore, no statistically significant association was found between *STAT4* (rs10181656, rs7574865, rs7601754, and rs10168266) in the early AMD, exudative AMD, and control groups among subjects aged > 65 to ≤75 years (Table 12).

The binomial logistic regression analysis of *STAT4* (rs10181656, rs7574865, rs7601754, and rs10168266) in the early AMD, exudative AMD, and control groups in the >75-year-old subjects did not reveal any statistically significant association (Table 13).

**Figure 1:**
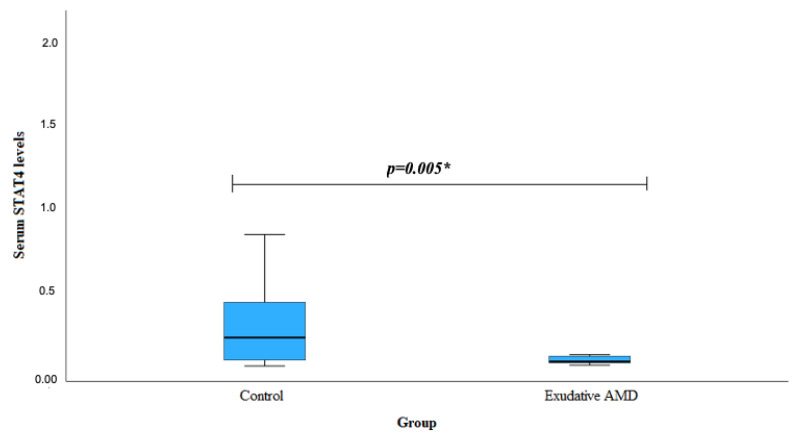
Serum levels of STAT4 in the control group and patients with exudative AMD. AMD: age-related macular degeneration; STAT4: signal transducer and activator of transcription 4. * The Mann–Whitney U test was used.

Serum STAT4 levels were measured in patients with exudative AMD (n = 40) and in the control group (n = 40). We found that exudative AMD patients had lower STAT4 serum levels when compared to the control group (median (IQR): 0.118 (0.042) vs. 0.262 (0.385), *p* = 0.005). The results are shown in Figure 1.

**Figure 2:**
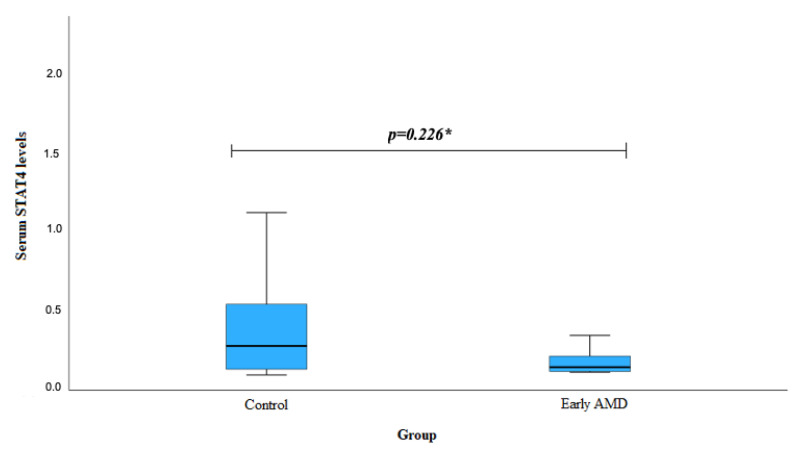
Serum levels of STAT4 in the control group and in patients with early AMD. AMD: age-related macular degeneration; STAT4: signal transducer and activator of transcription 4. * Student’s *t*-test was used.

However, no statistically significant differences were observed in the analysis of STAT4 levels between the early AMD and control groups (mean (std. deviation): 0.164 (0.068) vs. 0.859 (2.122), *p* = 0.226) (Figure 2).

A comparison of the serum STAT4 levels among different genotypes for selected single nucleotide polymorphisms was performed. The exudative AMD patients with at least one G allele of the *STAT4* rs10181656 had lower serum STAT4 levels than the control group subjects (*p* = 0.011). Also, the exudative AMD patients with at least one T allele of *STAT4* rs10168266 had lower serum STAT4 levels than the control group subjects (*p* = 0.039) (Table 14).

## 4. Discussion

We performed a study investigating the associations of the single nucleotide polymorphisms rs10181656, rs7574865, rs7601754, and rs10168266 of the *STAT4* gene with AMD. A total of 500 subjects participated in the study: 150 with early AMD, 150 with exudative AMD, and 200 healthy subjects. As far as we are aware, no scientific studies have been conducted to investigate the impact of these polymorphisms on AMD. The scientific literature states that STAT4 is involved in the pathogenesis of various autoimmune and inflammatory diseases, and the *STAT4* polymorphisms rs10181656, rs7574865, rs7601754, and rs10168266 are associated with various autoimmune diseases, such as rheumatoid arthritis (RA), systemic lupus erythematosus (SLE), and systemic sclerosis (SS) [20].

The analysis of the genotype and allele distribution of the single nucleotide polymorphisms rs10181656, rs7574865, rs7601754, and rs10168266 of *STAT4* did not reveal statistically significant data in our research. There are few studies on the *STAT4* rs10181656 SNP in the scientific literature databases. Scientific databases state that this SNP is associated with RA, SS, and SLE [20,21,22]. In a 2017–2018 study conducted in Iran, the *STAT4* rs10181656 polymorphism was strongly associated with RA risk (*p* = 0.007), but no association was found with SS [21]. The *STAT4* rs10181656 polymorphism has been associated with autoimmune diseases such as rheumatoid arthritis, systemic sclerosis, and systemic lupus erythematosus [21,22]. Studies investigating the association of the *STAT4* rs10181656 SNP with the above diseases can be found in the literature. One such study was conducted in Mexico and included 869 Mexican subjects, including 415 with RA, 128 with SLE, and 326 healthy controls. After investigating the association between *STAT4* rs7574865G/T polymorphism and the mentioned diseases, the *STAT4* rs7574865 G/T genotype was found to be associated with both RA and SLE risk [23].

In 2016, Greek researchers published a study that found that the GG genotype and the G allele of *STAT4* rs10181656 were significantly associated with the occurrence of psoriatic arthritis [24]. Ni Yan and coauthors investigated the association of *STAT4* polymorphisms with autoimmune thyroid diseases. The study, published in 2014, concluded that the *STAT4* rs7574685 T allele and the *STAT4* rs10181656 G allele were statistically significantly associated with the occurrence of thyroid autoimmune diseases such as Graves and Hashimototis in the Chinese Han population [25]. Most studies conducted on the *STAT4* rs7574865 SNP investigated the association of this polymorphism with RA and SLE. EnPeng GU and coauthors conducted a study systematizing data from 28 case–control studies examining the association between the *STAT4* rs7574865 polymorphism and RA. The results showed that the *STAT4* rs7574865 TT genotype, GT+TT genotype, and T allele were significantly associated with RA in European, Asian, South American, and African groups [26]. Junfeng Zheng and co-authors conducted a systematic review study, in 2013, which showed that the *STAT4* rs7574865 SNP is associated with three autoimmune diseases, RA, SS, and SLE, and that the *STAT4* rs7574865 T allele increases the probability of disease [20]. According to the results of a study by Ya-ling Liang and co-authors, the *STAT4* rs7574865 SNP is not only associated with RA, SS, and SLE but also slightly associated with the risk of type 1 diabetes, juvenile idiopathic arthritis (JIA), and ulcerative colitis (UC) [27]. A study by Hui Yuan and co-authors reports similar findings to the studies previously discussed: the *STAT4* rs7574865 polymorphism is statistically significantly associated with SLE in European and Asian groups. Still, no statistically significant data show that the *STAT4* rs7601754 SNP is associated with SLE, although the authors do not rule out this possibility [28]. The *STAT4* rs10168266 SNP is also associated with SLE risk. The association of the *STAT4* rs10168266 SNP with SLE is confirmed by a study conducted by Malaysian researchers, which reported that the *STAT4* rs10168266 SNP is significantly associated with the development of SLE in the Malaysian population [29]. A total of 790 Malaysian citizens participated in the study, of which 360 were SLE patients and 430 were healthy controls.

The *STAT4* polymorphism rs7574865 has been associated with autoimmune and liver diseases, such as hepatocellular carcinoma (HCC), chronic hepatitis B (CHB), and liver cirrhosis (LC) [30,31]. In 2022, a study was published in the PubMed database that was conducted among a Chinese Han population of 3151 subjects, of whom 968 had chronic hepatitis B, 316 had liver cirrhosis, and 1021 had hepatocellular carcinoma. The control group consisted of 846 healthy subjects. The research results suggest that the GG genotype of the *STAT4* polymorphism rs7574865 is significantly associated with the risk of HCC, LC, and CHB [30]. Gao Wenyan and co-authors published a study, in 2019, that aimed to systematically examine the association of the *STAT4* rs7574865 SNP with RA, including across ethnic groups. The systematic data showed that the T allele of the *STAT4* rs7574865 polymorphism is associated with the risk of RA in European and Asian populations but in groups of individuals aged 50–60 years on average [32]. The previously mentioned SNP (i.e., rs7574865) is also associated with systemic lupus erythematosus. The analysis by Jia-Min Wang and co-authors aimed to determine the associations of *STAT4* polymorphisms rs10168266 and rs7574865 with SLE risk. In the aforementioned study, the genotypes TT and CT of the *STAT4* polymorphisms rs10168266 and rs7574865 were found to be associated with SLE risk [33]. In the literature, rs7574865 is also associated with type 1 diabetes. The T allele and GT genotype of the *STAT4* rs7574865 polymorphism were statistically significantly associated with type 1 diabetes in Egyptian study patients [34]. In the scientific literature, there are few studies on the *STAT4* polymorphism rs7601754. One study in the PubMed database examined the associations of the *STAT4* rs7574865 and the *STAT4* rs7601754 SNP with SLE. The results presented stated that the mentioned *STAT4* rs7574865 polymorphism was associated with an increased risk of SLE, whereas the *STAT4* rs7601754 polymorphism was associated with a reduced risk of SLE [35].

There are also very few studies on the *STAT4* rs10168266 SNP. A literature review revealed that this polymorphism has been studied in the scientific literature databases mainly in the search for associations with SLE. A study conducted by Japanese researchers on 308 SLE patients and 306 control patients concluded that the *STAT4* polymorphisms rs7574865, rs11889341, and the aforementioned rs10168266 were associated with SLE risk. It was also found that the association between the above polymorphism and SLE was higher in the Japanese population than in the European or American populations [36]. Studies conducted by researchers associate single nucleotide polymorphisms of the *STAT4* gene rs10181656, rs7574865, rs7601754, and rs10168266 with various autoimmune diseases.

In our study, we investigated the association between the *STAT4* rs10181656, rs7574865, rs7601754, and rs10168266 single nucleotide polymorphisms (SNPs) and AMD. Contrary to our expectations, no significant associations were found, but it is important to note that these specific *STAT4* gene variants have not previously been studied, making definitive conclusions challenging.

Interestingly, our analysis of serum STAT4 levels revealed noteworthy findings. Exudative AMD patients carrying at least one G allele of *STAT4* rs10181656 exhibited significantly lower serum STAT4 levels than subjects in the control group (*p* = 0.011). Similarly, those with at least one T allele of *STAT4* rs10168266 had lower serum STAT4 levels than the control group subjects (*p* = 0.039). This observation suggests a potential link between specific STAT4 genotypes and decreased serum STAT4 levels in individuals with exudative AMD. Moreover, our study demonstrated a broader significance by revealing significantly lower overall serum STAT4 levels in exudative AMD patients compared to the control group (*p* = 0.005). This implies a potential association between decreased STAT4 levels and the presence of exudative AMD. However, no significant differences were observed in the STAT4 levels between the early AMD and control groups (*p* = 0.226). This suggests a nuanced role of STAT4, specifically implicated in the exudative stage of AMD, indicating a distinct association of STAT4 with different phases of AMD.

These findings not only contribute to our understanding of the genetic and serum level factors associated with AMD but also highlight the need for further exploration of the role of STAT4 in the context of different AMD stages.

While shedding light on the potential link between *STAT4* gene polymorphisms and AMD, our study has notable limitations. The existing literature lacks a definitive consensus on the role of *STAT4* in AMD pathogenesis, introducing an element of uncertainty. We acknowledge the omission of environmental factors, such as chronic light damage and aging, in our focus. Additionally, the use of serum STAT4 levels as a proxy for retinal tissue expression presents a limitation, prompting consideration for direct assessments in future research. The modest sample size, though offering initial insight, underscores the need for larger cohorts to validate our findings. Despite these limitations, our study contributes to the ongoing discourse on AMD, and we are committed to refining our approach in future investigations for a more comprehensive understanding.

## Figures and Tables

**Table 1:** Demographic characteristics.

Characteristics		Group			*p*-Value
		Early AMD *n* = 150	Exudative AMD *n* = 150	Control *n* = 200	
Gender	Women, N (%)	75 (50)	75 (50)	100 (50)	1 * 1 **
	Men, N (%)	75 (50)	75 (50)	100 (50)	
Interquartile range (IQR)		71 (11)	72.5 (11)	71 (4)	0.726 * 0.152 **

AMD—age-related macular degeneration. * Early AMD vs. control group. ** Exudative AMD vs. control group.

**Table 2:** Frequencies of *STAT4* (rs10181656, rs7574865, rs7601754, and rs10168266) genotypes and alleles in the early AMD and control groups.

SNP	Genotype/Allele	Group		*p*-Value
		Control, N (%)	Early AMD, N (%)	
*STAT4* rs10181656	CC	118 (59.0)	90 (60.0)	0.672
	CG	65 (32.5)	51 (34.0)	
	GG	17 (8.5)	9 (6.0)	
	Total:	200	150	
	Allele:			
	C	301 (75.25)	231 (77.0)	
	G	99 (24.75)	69 (23.0)	0.592
*STAT4* rs7574865	GG	118 (59.0)	92 (61.3)	0.670
	GT	65 (32.5)	49 32.7)	
	TT	17 (8.5)	9 (6.0)	
	Total:	200	150	
	Allele:			
	G	301 (75.25)	233 (77.67)	
	T	99 (24.75)	67 (22.33)	0.457
*STAT4* rs7601754	AA	150 (75.0)	113 (75.3)	0.902
	GA	46 (23.0)	33 (22.0)	
	GG	4 (2.0)	4 (2.7)	
	Total:	200	150	
	Allele:			
	A	346 (86.5)	259 (86.34)	
	G	54 (13.5)	41 (13.66)	0.949
*STAT4* rs10168266	CC	133 (66.5)	100 (66.7)	0.852
	CT	58 (29.0)	45 (30.0)	
	TT	9 (4.5)	5 (3.3)	
	Total:	200	150	
	Allele:			
	C	324 (81.0)	245 (81.67)	
	T	76 (19.0)	55 (18.33)	0.823

*p*—Significance level and Bonferroni-corrected significance level when *p* < 0.05/4; SNP—single nucleotide polymorphism; AMD—age-related macular degeneration.

**Table 3:** Frequencies of the *STAT4* (rs10181656, rs7574865, rs7601754, and rs10168266) genotypes and alleles in the exudative AMD and control groups.

SNP	Genotype/Allele	Group		*p*-Value
		Control, N (%)	Exudative AMD, N (%)	
*STAT4* rs10181656	CC	118 (59.0)	80 (53.3)	0.568
	CG	65 (32.5)	56 (37.3)	
	GG	17 (8.5)	14 (9.3)	
	Total:	200	150	
	Allele:			
	C	301 (75.25)	216 (72.0)	
	G	99 (24.75)	84 (28.0)	0.333
*STAT4* rs7574865	GG	118 (59.0)	80 (53.3)	0.568
	GT	65 (32.5)	56 (37.3)	
	TT	17 (8.5)	14 (9.3)	
	Total:	200	150	
	Allele:			
	G	301 (75.25)	216 (72.0)	
	T	99 (24.75)	84 (28.0)	0.333
*STAT4* rs7601754	AA	150 (75.0)	111 (74.0)	0.529
	GA	46 (23.0)	38 (25.3)	
	GG	4 (2.0)	1 (0.7)	
	Total:	200	150	
	Allele:			
	A	346 (86.5)	260 (86.67)	
	G	54 (13.5)	40 (13.33)	0.949
*STAT4* rs10168266	CC	133 (66.5)	98 (65.3)	0.674
	CT	58 (29.0)	42 (28.0)	
	TT	9 (4.5)	10 (6.7)	
	Total:	200	150	
	Allele:			
	C	324 (81.0)	238 (79.34)	
	T	76 (19.0)	62 (20.96)	0.583

*p*—Significance level and Bonferroni-corrected significance level when *p* < 0.05/4; SNP—single nucleotide polymorphism; AMD—age-related macular degeneration.

**Table 4:** Binomial logistic regression analysis of *STAT4* (rs10181656, rs7574865, rs7601754, and rs10168266) in the early AMD and control groups.

Model	Genotype/Allele	OR (95% CI)	*p*-Value	AIC
*STAT4* rs10181656				
Codominant	CG vs. CC GG vs. CC	1.029 (0.651–1.626) 0.694 (0.296–1.629)	0.904 0.402	481.227
Dominant	CG+GG vs. CC	0.959 (0.623–1.477)	0.850	480.000
Recessive	GG vs. CC+CG	0.687 (0.297–1.587)	0.380	479.241
Overdominant	CG vs. CC+GG	1.070 (0.683–1.676)	0.768	479.949
Additive	C	0.915 (0.653–1.283)	0.608	479.771
*STAT4* rs7574865				
Codominant	GT vs. GG TT vs. GG	0.967 (0.610–1.532) 0.679 (0.289–1.593)	0.886 0.374	481.221
Dominant	GT+TT vs. GG	0.907 (0.588–1.399)	0.659	479.841
Recessive	TT vs. GG+GT	0.687 (0.297–1.587)	0.380	479.241
Overdominant	GT vs. GG+TT	1.008 (0.641–1.583)	0.974	480.035
Additive	G	0.885 (0.631–1.241)	0.478	479.530
*STAT4* rs7601754				
Codominant	GA vs. AA GG vs. AA	0.952 (0.572–1.585) 1.327 (0.325–5.422)	0.851 0.693	481.831
Dominant	GA+GG vs. AA	0.982 (0.602–1.604)	0.943	480.031
Recessive	GG vs. AA+GA	1.342 (0.330–5.457)	0.681	479.867
Overdominant	GA vs. AA+GG	0.944 (0.568–1.569)	0.825	479.987
Additive	A	1.014 (0.660–1.556)	0.950	480.032
*STAT4* rs10168266				
Codominant	CT vs. CC TT vs. CC	1.032 (0.646–1.648) 0.739 (0.240–2.273)	0.895 0.598	481.709
Dominant	CT+TT vs. CC	0.993 (0.634–1.555)	0.974	480.035
Recessive	TT vs. CC+CT	0.732 (0.240–2.230)	0.583	479.727
Overdominant	CT vs. CC+TT	1.049 (0.660–1.669)	0.839	479.994
Additive	C	0.958 (0.656–1.399)	0.826	479.987

*p*—Significance level and Bonferroni-corrected significance level when *p* < 0.05/4; OR—odds ratio; CI—confident interval; AIC—Akaike information criteria.

**Table 5:** Binomial logistic regression analysis of *STAT4* (rs10181656, rs7574865, rs7601754, and rs10168266) in the exudative AMD and control groups.

Model	Genotype/Allele	OR (95% CI)	*p*-Value	AIC
*STAT4* rs10181656				
Codominant	CG vs. CC GG vs. CC	1.271 (0.805–2.006) 1.215 (0.567–2.603)	0.303 0.617	480.904
Dominant	CG+GG vs. CC	1.259 (0.822–1.930)	0.290	478.916
Recessive	GG vs. CC+CG	1.108 (0.528–2.326)	0.786	479.962
Overdominant	CG vs. CC+GG	1.237 (0.794–1.929)	0.347	479.153
Additive	C	1.164 (0.842–1.608)	0.357	479.189
*STAT4* rs7574865				
Codominant	GT vs. GG TT vs. GG	1.271 (0.805–2.006) 1.215 (0.567–2.603)	0.303 0.617	480.904
Dominant	GT+TT vs. GG	1.259 (0.822–1.930)	0.290	478.916
Recessive	TT vs. GG+GT	1.108 (0.528–2.326)	0.786	479.962
Overdominant	GT vs. GG+TT	1.237 (0.794–1.929)	0.347	479.153
Additive	G	1.164 (0.842–1.608)	0.357	479.189
*STAT4* rs7601754				
Codominant	GA vs. AA GG vs. AA	1.116 (0.681–1.831) 0.338 (0.037–3.064)	0.663 0.335	480.663
Dominant	GA+GG vs. AA	1.054 (0.649–1.713)	0.832	479.991
Recessive	GG vs. AA+GA	0.329 (0.036–2.973)	0.322	478.853
Overdominant	GA vs. AA+GG	1.136 (0.693–1.861)	0.613	479.781
Additive	A	0.985 (0.630–1.540)	0.948	480.031
*STAT4* rs10168266				
Codominant	CT vs. CC TT vs. CC	0.983 (0.611–1.581) 1.508 (0.590–3.851)	0.943 0.391	481.256
Dominant	CT+TT vs. CC	1.053 (0.674–1.646)	0.820	479.984
Recessive	TT vs. CC+CT	1.516 (0.600–3.829)	0.379	479.261
Overdominant	CT vs. CC+TT	0.952 (0.595–1.522)	0.838	479.994
Additive	C	1.100 (0.769–1.574)	0.601	479.762

*p*—Significance level and Bonferroni-corrected significance level when *p* < 0.05/4; OR—odds ratio; CI—confident interval; AIC—Akaike information criteria.

**Table 6:** Frequencies of *STAT4* (rs10181656, rs7574865, rs7601754, and rs10168266) genotypes and alleles in the early AMD and control groups by gender.

SNP	Genotype/Allele	Women		*p*-Value	Men		*p*-Value
		Control, N (%)	Early AMD, N (%)		Control, N (%)	Early AMD, N (%)	
*STAT4* rs10181656	CC CG GG Allele: C G	58 (58.0) 33 (33.0) 9 (9.0) 149 (74.5) 51 (25.5)	48 (64.0) 22 (29.3) 5 (6.7) 118 (78.67) 32 (21.33)	0.694 0.364	60 (60.0) 32 (32.0) 8 (8.0) 152 (76.0) 48 (24.0)	42 (56.0) 29 (38.7) 4 (5.3) 113 (75.34) 37 (24.66)	0.574 0.886
*STAT4* rs7574865	GG GT TT Allele: G T	58 (58.0) 33 (33.0) 9 (9.0) 149 (74.5) 51 (25.5)	48 (64.0) 21 (28.0) 6 (8.0) 117 (78.0) 33 (22.0)	0.722 0.448	60 (60.0) 32 (32.0) 8 (8.0) 152 (76.0) 48 (24.0)	44 (58.7) 28 (37.3) 3 (4.0) 116 (77.34) 34 (22.66)	0.482 0.771
*STAT4* rs7601754	AA GAGG Allele: A G	73 (73.0) 25 (25.0) 2 (2.0) 171 (85.5) 29 (14.5)	50 (66.7) 21 (28.0) 4 (5.3) 121 (80.67) 29 (19.33)	0.411 0.229	77 (77.0) 21 (21.0) 2 (2.0) 175 (87.5) 25 (12.5)	63 (84.0) 12 (16.0) 0 (0.0) 138 (92.0) 12 (8.0)	0.312 0.175
*STAT4* rs10168266	CC CT TT Allele: C T	62 (62.0) 32 (32.0) 6 (6.0) 156 (78.0) 44 (22.0)	50 (66.7) 22 (29.3) 3 (4.0) 122 (81.33) 28 (18.67)	0.749 0.445	71 (71.0) 26 (26.0) 3 (3.0) 168 (84.0) 32 (16.0)	50 (66.7) 23 (30.7) 2 (2.7) 123 (82.0) 27 (18.0)	0.792 0.621

*p*—Significance level and Bonferroni-corrected significance level when *p* < 0.05/4; SNP—single nucleotide polymorphism; AMD—age-related macular degeneration.

**Table 7:** Frequencies of *STAT4* (rs10181656, rs7574865, rs7601754, and rs10168266) genotypes and alleles in the exudative AMD and control groups by gender.

SNP	Genotype/Allele	Women		*p*-Value	Men		*p*-Value
		Control, N (%)	Exudative AMD, N (%)		Control, N (%)	Exudative AMD, N (%)	
*STAT4* rs10181656	CC CG GG Allele: C G	58 (58.0) 33 (33.0) 9 (9.0) 149 (74.5) 51 (25.5)	37 (49.3) 31 (41.3) 7 (9.3) 105 (70.0) 45 (30.0)	0.494 0.350	60 (60.0) 32 (32.0) 8 (8.0) 152 (76.0) 48 (24.0)	43 (57.3) 25 (33.3) 7 (9.3) 111 (74.0) 39 (26.0)	0.921 0.668
*STAT4* rs7574865	GG GT TT Allele: G T	58 (58.0) 33 (33.0) 9 (9.0) 149 (74.5) 51 (25.5)	37 (49.3) 31 (41.3) 7 (9.3) 105 (70.0) 45 (30.0)	0.494 0.350	60 (60.0) 32 (32.0) 8 (8.0) 152 (76.0) 48 (24.0)	43 (57.3) 25 (33.3) 7 (9.3) 111 (74.0) 39 (26.0)	0.921 0.668
*STAT4* rs7601754	AA GA GG Allele: A G	73 (73.0) 25 (25.0) 2 (2.0) 171 (85.5) 29 (14.5)	54 (72.0) 21 (28.0) 0 (0) 129 (86.0) 21 (14.0)	0.438 0.895	77 (77.0) 21 (21.0) 2 (2.0) 175 (87.5) 25 (12.5)	57 (76.0) 17 (22.7) 1 (1.3) 131 (87.34) 19 (12.66)	0.918 0.963
*STAT4* rs10168266	CC CT TT Allele: C T	62 (62.0) 32 (32.0) 6 (6.0) 156 (78.0) 44 (22.0)	47 (62.7) 23 (30.7) 5 (6.7) 117 (78.0) 33 (22.0)	0.972 1	71 (71.0) 26 (26.0) 3 (3.0) 168 (84.0) 32 (16.0)	51 (68.0) 19 (25.3) 5 (6.7) 121 (80.67) 29 (19.33)	0.516 0.416

*p*—Significance level and Bonferroni-corrected significance level when *p* < 0.05/4; SNP—single nucleotide polymorphism; AMD—age-related macular degeneration.

**Table 8:** Binary logistic regression analysis of *STAT4* (rs10181656, rs7574865, rs7601754, and rs10168266) in the early AMD and control groups by gender.

Model	Genotype/Allele	OR (95% CI)	*p*-Value	AIC
Women				
*STAT4* rs10181656				
Codominant	CG vs. CC GG vs. CC	0.806 (0.416–1.561) 0.671 (0.211–2.137)	0.522 0.500	242.283
Dominant	CG+GG vs. CC	0.777 (0.419–1.439)	0.422	240.370
Recessive	GG vs. CC+CG	0.722 (0.232–2.251)	0.575	240.696
Overdominant	CG vs. CC+GG	0.843 (0.441–1.612)	0.605	240.750
Additive	C	0.814 (0.506–1.309)	0.395	240.285
*STAT4* rs7574865				
Codominant	GT vs. GG TT vs. GG	0.769 (0.394–1.499) 0.806 (0.268–2.424)	0.440 0.700	242.364
Dominant	GT+TT vs. GG	0.777 (0.419–1.439)	0.422	240.370
Recessive	TT vs. GG+GT	0.879 (0.299–2.587)	0.815	240.963
Overdominant	GT vs. GG+TT	0.790 (0.411–1.519)	0.479	240.513
Additive	G	0.845 (0.530–1.348)	0.481	240.516
*STAT4* rs7601754				
Codominant	GA vs. AA GG vs. AA	1.226 (0.620–2.427) 2.920 (0.515–16.555)	0.558 0.226	239.247
Dominant	GA+GG vs. AA	1.352 (0.704–2.595)	0.365	240.198
Recessive	GG vs. AA+GA	2.761 (0.492–15.488)	0.249	239.590
Overdominant	GA vs. AA+GG	1.167 (0.593–2.297)	0.656	240.819
Additive	A	1.392 (0.799–2.424)	0.242	239.646
*STAT4* rs10168266				
Codominant	CT vs. CC TT vs. CC	0.853 (0.441–1.647) 0.620 (0.148–2.604)	0.635 0.514	242.431
Dominant	CT+TT vs. CC	0.816 (0.436–1.528)	0.525	240.611
Recessive	TT vs. CC+CT	0.653 (0.158–2.699)	0.556	240.658
Overdominant	CT vs. CC+TT	0.882 (0.460–1.691)	0.706	240.875
Additive	C	0.822 (0.490–1.379)	0.458	240.461
Men				
*STAT4* rs10181656				
Codominant	CG vs. CC GG vs. CC	1.295 (0.684–2.452) 0.714 (0.202–2.527)	0.428 0.602	241.902
Dominant	CG+GG vs. CC	1.179 (0.643–2.162)	0.595	240.736
Recessive	GG vs. CC+CG	0.648 (0.188–2.238)	0.493	240.529
Overdominant	CG vs. CC+GG	1.340 (0.716–2.507)	0.360	240.182
Additive	C	1.035 (0.640–1.674)	0.888	240.998
*STAT4* rs7574865				
Codominant	GT vs. GG TT vs. GG	1.193 (0.630–2.261) 0.511 (0.128–2.038)	0.588 0.342	241.505
Dominant	GT+TT vs. GG	1.057 (0.575–1.944)	0.859	240.986
Recessive	TT vs. GG+GT	0.479 (0.123–1.871)	0.290	239.798
Overdominant	GT vs. GG+TT	1.266 (0.675–2.374)	0.462	240.478
Additive	G	0.931 (0.570–1.521)	0.776	240.936
*STAT4* rs7601754				
Codominant	GA vs. AA GG vs. AA	0.698 (0.319–1.529) -	0.369 -	239.941
Dominant	GA+GG vs. AA	0.638 (0.294–1.382)	0.254	239.683
Recessive	GG vs. AA+GA	-	-	-
Overdominant	GA vs. AA+GG	0.717 (0.328–1.567)	0.404	240.309
Additive	A	0.608 (0.294–1.259)	0.180	239.135
*STAT4* rs10168266				
Codominant	CT vs. CC TT vs. CC	1.256 (0.644–2.449) 0.947 (0.153–5.874)	0.503 0.953	242.553
Dominant	CT+TT vs. CC	1.224 (0.642–2.335)	0.539	240.642
Recessive	TT vs. CC+CT	0.886 (0.144–5.439)	0.896	241.001
Overdominant	CT vs. CC+TT	1.259 (0.648–2.445)	0.497	240.557
Additive	C	1.152 (0.656–2.023)	0.621	240.774

*p*—Significance level and Bonferroni-corrected significance level when *p* < 0.05/4; OR—odds ratio; CI—confident interval; AIC—Akaike information criteria.

**Table 9:** Binomial logistic regression analysis of *STAT4* (rs10181656, rs7574865, rs7601754, and rs10168266) in the exudative AMD and control groups by gender.

Model	Genotype/Allele	OR (95% CI)	*p*-Value	AIC
Women				
*STAT4* rs10181656				
Codominant	CG vs. CC GG vs. CC	1.473 (0.776–2.794) 1.219 (0.418–3.556)	0.236 0.717	241.608
Dominant	CG+GG vs. CC	1.418 (0.777–2.590)	0.255	239.721
Recessive	GG vs. CC+CG	1.041 (0.369–2.934)	0.940	241.012
Overdominant	CG vs. CC+GG	1.430 (0.769–2.660)	0.258	239.739
Additive	C	1.232 (0.781–1.942)	0.370	240.214
*STAT4* rs7574865				
Codominant	GT vs. GG TT vs. GG	1.473 (0.776–2.794) 1.219 (0.418–3.556)	0.236 0.717	241.608
Dominant	GT+TT vs. GG	1.418 (0.777–2.590)	0.255	239.721
Recessive	TT vs. GG+GT	1.041 (0.369–2.934)	0.940	241.012
Overdominant	GT vs. GG+TT	1.430 (0.769–2.660)	0.258	239.739
Additive	G	1.232 (0.781–1.942)	0.370	240.214
*STAT4* rs7601754				
Codominant	GA vs. AA GG vs. AA	1.136 (0.576–2.238) -	0.713 -	240.627
Dominant	GA+GG vs. AA	1.051 (0.538–2.055)	0.883	240.996
Recessive	GG vs. AA+GA	-	-	-
Overdominant	GA vs. AA+GG	1.167 (0.593–2.297)	0.656	240.819
Additive	A	0.957 (0.510–1.796)	0.891	240.999
*STAT4* rs10168266				
Codominant	CT vs. CC TT vs. CC	0.948 (0.492–1.828) 1.099 (0.316–3.821)	0.874 0.882	242.960
Dominant	CT+TT vs. CC	0.972 (0.524–1.803)	0.928	241.010
Recessive	TT vs. CC+CT	1.119 (0.328–3.815)	0.857	240.986
Overdominant	CT vs. CC+TT	0.940 (0.493–1.793)	0.851	240.982
Additive	C	1.000 (0.612–1.634)	1.000	241.018
Men				
*STAT4* rs10181656				
Codominant	CG vs. CC GG vs. CC	1.090 (0.567–2.095) 1.221 (0.412–3.622)	0.796 0.719	242.854
Dominant	CG+GG vs. CC	1.116 (0.608–2.050)	0.723	240.892
Recessive	GG vs. CC+CG	1.184 (0.409–3.423)	0.755	240.921
Overdominant	CG vs. CC+GG	1.062 (0.561–2.011)	0.852	240.983
Additive	C	1.099 (0.694–1.741)	0.687	240.856
*STAT4* rs7574865				
Codominant	GT vs. GG TT vs. GG	1.090 (0.567–2.095) 1.221 (0.412–3.622)	0.796 0.719	242.854
Dominant	GT+TT vs. GG	1.116 (0.608–2.050)	0.723	240.892
Recessive	TT vs. GG+GT	1.184 (0.409–3.423)	0.755	240.921
Overdominant	GT vs. GG+TT	1.062 (0.561–2.011)	0.852	240.983
Additive	G	1.099 (0.694–1.741)	0.687	240.856
*STAT4* rs7601754				
Codominant	GA vs. AA GG vs. AA	1.094 (0.529–2.259) 0.675 (0.060–7.632)	0.809 0.751	242.844
Dominant	GA+GG vs. AA	1.057 (0.522–2.141)	0.877	240.994
Recessive	GG vs. AA+GA	0.662 (0.059–7.442)	0.738	240.902
Overdominant	GA vs. AA+GG	1.103 (0.535–2.274)	0.791	240.948
Additive	A	1.015 (0.538–1.914)	0.963	241.016
*STAT4* rs10168266				
Codominant	CT vs. CC TT vs. CC	1.017 (0.509–2.033) 2.320 (0.530–10.151)	0.961 0.264	241.709
Dominant	CT+TT vs. CC	1.152 (0.602–2.206)	0.669	240.836
Recessive	TT vs. CC+CT	2.310 (0.534–9.984)	0.262	239.712
Overdominant	CT vs. CC+TT	0.966 (0.486–1.917)	0.920	241.008
Additive	C	1.231 (0.726–2.087)	0.440	240.423

*p*—Significance level and Bonferroni-corrected significance level when *p* < 0.05/4; OR—odds ratio; CI—confident interval; AIC—Akaike information criteria.

**Table 10:** Frequencies of *STAT4* (rs10181656, rs7574865, rs7601754, and rs10168266) genotypes and alleles in the early AMD, exudative AMD, and control groups by age.

SNP	Genotype/Allele	≤65 y/o			*p*-Value	>65 y/o–≤75 y/o			*p*-Value	>75 y/o			*p*-Value
		Control Group,	Early AMD	Exudative AMD		Control Group,	Early AMD	Exudative AMD		Control Group,	Early AMD	Exudative AMD	
		n (%)	n (%)	n (%)		n (%)	n (%)	n (%)		n (%)	n (%)	n (%)	
*rs10181656*	CC	13 (52.0)	15 (50.0)	17 (54.8)	0.965 (1) 0.754 (2)	89 (59.7)	48 (62.3)	32 (50.0)	0.493 (1) 0.411 (2)	16 (61.5)	27 (62.8)	31 (56.4)	0.797 (1) 0.447 (2)
	CG	10 (40.0)	12 (40.0)	10 (32.3)		48 (32.2)	26 (33.8)	25 (39.1)		7 (26.9)	13 (30.2)	21 (38.2)	
	GG	2 (8.0)	3 (10.0)	4 (12.9)		12 (8.1)	3 (3.9)	7 (10.9)		3 (11.5)	3 (7.0)	3 (5.5)	
	C	36 (72)	42 (70)	44 (71)	0.818 (1) 0.904 (2)	226 (75.8)	122 (79.2)	89 (69.5)	0.418 (1) 0.174 (2)	39 (75)	67 (77.9)	83 (75.5)	0.695 (1) 0.950 (2)
	G	14 (28)	18 (30)	18 (29)		72 (24.2)	32 (20.8)	39 (30.5)		13 (25)	19 (22.1)	27 (24.5)	
*rs7574865*	GG	13 (52.0)	17 (56.7)	17 (54.8)	0.938 (1) 0.754 (2)	89 (59.7)	48 (62.3)	31 (48.4)	0.725 (1) 0.277 (2)	16 (61.5)	27 (62.8)	32 (58.2)	0.797 (1) 0.291 (2)
	GT	10 (40.0)	11 (36.7)	10 (32.3)		48 (32.2)	25 (32.5)	25 (39.1)		7 (26.0)	13 (30.2)	21 (38.2)	
	TT	2 (8.0)	2 (7.7)	4 (12.9)		12 (8.1)	4 (5.2)	8 (12.5)		3 (11.5)	3 (7.0)	2 (3.6)	
	G	36 (72)	45 (75)	44 (71)	0.722 (1) 0.904 (2)	226 (75.8)	121 (78.6)	87 (68)	0.514 (1) 0.092 (2)	39 (75)	67 (77.9)	85 (77.3)	0.695 (1) 0.749 (2)
	T	14 (28)	15 (25)	18 (29)		72 (24.2)	33 (21.4)	41 (32)		13 (25)	19 (22.1)	25 (22.7)	
*rs7601754*	AA	19 (76.0)	18 (60.0)	21 (67.7)	0.453 (1) 0.342 (2)	113 (75.8)	57 (74.0)	51 (79.7)	0.857 (1) 0.486 (2)	18 (69.2)	38 (88.4)	39 (70.9)	0.060 (1) 0.757 (2)
	GA	5 (20.0)	10 (33.3)	10 (32.3)		33 (22.1)	19 (24.7)	13 (20.3)		8 (30.8)	4 (9.3)	15 (27.3)	
	GG	1 (4.0)	2 (6.7)	0 (0)		3 (2.0)	1 (1.3)	0 (0)		0 (0)	1 (2.3)	1 (1.8)	
	A	43 (86)	46 (76.7)	52 (83.9)	0.215 (1) 0.755 (2)	259 (86.9)	133 (86.4)	115 (89.8)	0.870 (1) 0.397 (2)	44 (84.6)	80 (93)	93 (84.6)	0.113 (1) 0.991 (2)
	G	7 (14)	14 (23.3)	10 (16.1)		39 (13.1)	21 (13.6)	13 (10.2)		8 (15.4)	6 (7)	17 (15.4)	
*rs10168266*	CC	14 (56.0)	17 (56.7)	18 (58.1)	0.956 (1) 0.972 (2)	101 (67.8)	53 (68.8)	38 (59.4)	0.528 (1) 0.234 (2)	18 (69.2)	30 (69.8)	42 (76.1)	0.934 (1) 0.777 (2)
	CT	9 (36.0)	10 (33.3)	11 (35.5)		42 (28.2)	23 (29.9)	20 (31.3)		7 (26.9)	12 (27.9)	11 (20.0)	
	TT	2 (8.0)	3 (10.0)	2 (6.5)		6 (4.0)	1 (1.3)	6 (9.4)		1 (3.8)	1 (2.3)	2 (3.6)	
	C	37 (74)	44 (73.3)	47 (75.8)	0.937 (1) 0.826 (2)	244 (81.9)	129 (83.8)	96 (75)	0.617 (1) 0.105 (2)	43 (82.7)	72 (83.7)	95 (86.4)	0.875 (1) 0.539 (2)
	T	13 (26)	16 (26.7)	15 (24.2)		54 (18.1)	25 (16.2)	32 (25)		9 (17.3)	14 (16.3)	15 (13.6)	

*p*—Significance level and Bonferroni-corrected significance level when *p* < 0.05/4; SNP—single nucleotide polymorphism; AMD—age-related macular degeneration. (1)—Early AMD vs. control group; (2)—exudative AMD vs. control group.

**Table 11:** Binomial logistic regression analysis of *STAT4* (rs10181656, rs7574865, rs7601754, and rs10168266) in the early AMD, exudative AMD, and control groups in the ≤65-year-old subjects.

≤65 y/o				
Model	Genotype/Allele	OR (95% CI) *	*p*-Value	AIC
Early AMD				
rs10181656				
Codominant	CG vs. CC	1.040 (0.339–3.190)	0.945	79.720
	GG vs. CC	1.300 (0.187–9.021)	0.791	
Dominant	CG+GG vs. CC	1.083 (0.375–3.133)	0.883	77.769
Recessive	GG vs. CG+CC	1.278 (0.196–8.321)	0.798	77.724
Overdominant	CG vs. CC+GG	1.000 (0.338–2.955)	1.000	77.791
Additive	C	1.099 (0.486–2.486)	0.821	77.740
rs7574865				
Codominant	GT vs. GG	0.841 (0.274–2.579)	0.762	79.664
	TT vs. GG	0.765 (0.095–6.175)	0.801	
Dominant	GT+TT vs. GG	0.828 (0.285–2.406)	0.729	77.671
Recessive	TT vs. GG+GT	0.821 (0.107–6.293)	0.850	77.755
Overdominant	GT vs. GG+TT	0.868 (0.292–2.587)	0.800	77.727
Additive	G	0.859 (0.369–1.999)	0.725	77.667
rs7601754				
Codominant	GA vs. AA	2.111 (0.604–7.385)	0.242	78.180
	GG vs. AA	2.111 (0.176–25.349)	0.556	
Dominant	GA+GG vs. AA	2.111 (0.653–6.823)	0.212	76.180
Recessive	GG vs. AA+GA	1.714 (0.146–20.097)	0.668	77.598
Overdominant	GA vs. AA+GG	2.000 (0.579–6.908)	0.273	76.547
Additive	A	1.766 (0.673–4.634)	0.248	76.375
rs10168266				
Codominant	CT vs. CC	0.915 (0.291–2.876)	0.879	79.701
	TT vs. CC	1.235 (0.180–8.459)	0.830	
Dominant	CT+TT vs. CC	0.973 (0.334–2.838)	0.960	77.789
Recessive	TT vs. CC+CT	1.278 (0.196–8.321)	0.798	77.724
Overdominant	CT vs. CC+TT	0.889 (0.291–2.711)	0.836	77.748
Additive	C	1.031 (0.459–2.317)	0.940	77.785
Exudative AMD				
rs10181656				
Codominant	CG vs. CC	0.765 (0.246–2.381)	0.643	80.418
	GG vs. CC	1.529 (0.242–9.674)	0.652	
Dominant	CG+GG vs. CC	0.892 (0.310–2.566)	0.832	78.944
Recessive	GG vs. CG+CC	1.704 (0.286–10.165)	0.559	78.633
Overdominant	CG vs. CC+GG	0.714 (0.238–2.143)	0.548	78.628
Additive	C	1.046 (0.480–2.279)	0.910	78.976
rs7574865				
Codominant	GT vs. GG	0.765 (0.246–2.381)	0.643	80.418
	TT vs. GG	1.529 (0.242–9.674)	0.652	
Dominant	GT+TT vs. GG	0.892 (0.310–2.566)	0.832	78.944
Recessive	TT vs. GG+GT	1.704 (0.286–10.165)	0.559	78.633
Overdominant	GT vs. GG+TT	0.714 (0.238–2.143)	0.548	78.628
Additive	G	1.046 (0.480–2.279)	0.910	78.976
rs7601754				
Codominant	GA vs. AA	1.810 (0.524–6.253)	0.349	78.447
	GG vs. AA	-	-	
Dominant	GA+GG vs. AA	1.508 (0.460–4.943)	0.498	78.522
Recessive	GG vs. AA+GA	-	-	-
Overdominant	GA vs. AA+GG	1.905 (0.553–6.555)	0.307	77.909
Additive	A	1.190 (0.408–3.467)	0.750	78.886
rs10168266				
Codominant	CT vs. CC	0.951 (0.309–2.926)	0.930	80.931
	TT vs. CC	0.778 (0.097–6.230)	0.813	
Dominant	CT+TT vs. CC	0.919 (0.317–2.664)	0.877	78.964
Recessive	TT vs. CC+CT	0.793 (0.104–6.069)	0.823	78.939
Overdominant	CT vs. CC+TT	0.978 (0.326–2.935)	0.968	78.987
Additive	C	0.912 (0.394–2.112)	0.830	78.942

*p*—Significance level and Bonferroni-corrected significance level when *p* < 0.05/4; OR—odds ratio; CI—confident interval; AIC—Akaike information criteria. * 95% confidence interval (CI) of the mean is a range with an upper and lower number calculated.

**Table 12:** Binomial logistic regression analysis of *STAT4* (rs10181656, rs7574865, rs7601754, and rs10168266) in the early AMD, exudative AMD, and control groups in the >65–≤75-year-old subjects.

>65 y/o–≤75 y/o				
Model	Genotype/Allele	OR (95% CI) *	*p*-Value	AIC
Early AMD				
rs10181656				
Codominant	CG vs. CC	1.004 (0.555–1.816)	0.989	292.420
	GG vs. CC	0.464 (0.125–1.723)	0.251	
Dominant	CG+GG vs. CC	0.896 (0.509–1.577)	0.704	291.815
Recessive	GG vs. CG+CC	0.463 (0.127–1.692	0.244	290.420
Overdominant	CG vs. CC+GG	1.073 (0.598–1.924)	0.814	291.904
Additive	C	0.834 (0.529–1.316)	0.436	291.342
rs7574865				
Codominant	GT vs. GG	0.966 (0.531–1.755)	0.909	293.285
	TT vs. GG	0.618 (0.189–2.021)	0.426	
Dominant	GT+TT vs. GG	0.896 (0.509–1.577)	0.704	291.815
Recessive	TT vs. GG+GT	0.626 (0.195–2.009)	0.431	291.298
Overdominant	GT vs. GG+TT	1.012 (0.562–1.821)	0.969	291.958
Additive	G	0.867 (0.554–1.357)	0.534	291.567
rs7601754				
Codominant	GA vs. AA	1.141 (0.597–2.182)	0.689	293.644
	GG vs. AA	0.661 (0.067–6.496)	0.722	
Dominant	GA+GG vs. AA	1.101 (0.585–2.073)	0.765	291.871
Recessive	GG vs. AA+GA	0.640 (0.065–6.261)	0.702	291.803
Overdominant	GA vs. AA+GG	1.152 (0.603–2.198)	0.669	291.778
Additive	A	1.049 (0.593–1.854)	0.871	291.933
rs10168266				
Codominant	CT vs. CC	1.044 (0.568–1.916)	0.891	292.491
	TT vs. CC	0.318 (0.037–2.707)	0.294	
Dominant	CT+TT vs. CC	0.953 (0.527–1.723)	0.873	291.934
Recessive	TT vs. CC+CT	0.314 (0.037–2.653)	0.287	290.510
Overdominant	CT vs. CC+TT	1.085 (0.593–1.986)	0.791	291.890
Additive	C	0.876 (0.521–1.473)	0.617	291.707
Exudative AMD				
rs10181656				
Codominant	CG vs. CC	1.449 (0.771–2.720)	0.249	262.633
	GG vs. CC	1.622 (0.587–4.481)	0.351	
Dominant	CG+GG vs. CC	1.483 (0.823–2.674)	0.190	260.678
Recessive	GG vs. CG+CC	1.402 (0.525–3.743)	0.500	261.955
Overdominant	CG vs. CC+GG	1.349 (0.734–2.479)	0.335	261.476
Additive	C	1.333 (0.860–2.067)	0.199	260.763
rs7574865				
Codominant	GT vs. GG	1.495 (0.794–2.816)	0.213	261.862
	TT vs. GG	1.914 (0.716–5.118)	0.196	
Dominant	GT+TT vs. GG	1.579 (0.876–2.847)	0.129	260.086
Recessive	TT vs. GG+GT	1.631 (0.633–4.205)	0.311	261.405
Overdominant	GT vs. GG+TT	1.349 (0.734–2.479)	0.335	261.476
Additive	G	1.419 (0.920–2.190)	0.113	259.911
rs7601754				
Codominant	GA vs. AA	0.873 (0.424–1.797)	0.712	262.097
	GG vs. AA	-	-	
Dominant	GA+GG vs. AA	0.800 (0.391–1.636)	0.541	262.017
Recessive	GG vs. AA+GA	-	-	-
Overdominant	GA vs. AA+GG	0.896 (0.436–1.843)	0.765	262.308
Additive	A	0.749 (0.384–1.462)	0.397	261.651
rs10168266				
Codominant	CT vs. CC	1.266 (0.661–2.425)	0.478	261.680
	TT vs. CC	2.658 (0.807–8.750)	0.108	
Dominant	CT+TT vs. CC	1.440 (0.786–2.638)	0.238	261.018
Recessive	TT vs. CC+CT	2.466 (0.764–7.960)	0.131	260.179
Overdominant	CT vs. CC+TT	1.158 (0.612–2.191)	0.652	262.196
Additive	C	1.454 (0.902–2.344)	0.124	260.067

*p*—Significance level and Bonferroni-corrected significance level when *p* < 0.05/4; OR—odds ratio; CI—confident interval; AIC—Akaike information criteria. * 95% confidence interval (CI) of the mean is a range with an upper and lower number calculated.

**Table 13:** Binomial logistic regression analysis of *STAT4* (rs10181656, rs7574865, rs7601754, and rs10168266) in the early AMD, exudative AMD, and control groups in the >75-year-old subjects.

>75 y/o				
Model	Genotype/Allele	OR (95% CI) *	*p*-Value	AIC
Early AMD				
rs10181656				
Codominant	CG vs. CC	1.101 (0.364–3.331)	0.865	94.981
	GG vs. CC	0.593 (0.107–3.295)	0.550	
Dominant	CG+GG vs. CC	0.948 (0.348–2.586)	0.917	93.412
Recessive	GG vs. CG+CC	0.575 (0.107–3.087)	0.519	93.010
Overdominant	CG vs. CC+GG	1.176 (0.398–3.477)	0.769	93.336
Additive	C	0.873 (0.415–1.833)	0.719	93.294
rs7574865				
Codominant	GT vs. GG	1.101 (0.364–3.331)	0.865	94.981
	TT vs. GG	0.593 (0.107–3.295)	0.550	
Dominant	GT+TT vs. GG	0.948 (0.348–2.586)	0.917	93.412
Recessive	TT vs. GG+GT	0.575 (0.107–3.087)	0.519	93.010
Overdominant	GT vs. GG+TT	1.176 (0.398–3.477)	0.769	93.336
Additive	G	0.873 (0.415–1.833)	0.719	93.294
rs7601754				
Codominant	GA vs. AA	0.237 (0.063–0.891)	0.033	89.606
	GG vs. AA	-	-	
Dominant	GA+GG vs. AA	0.296 (0.085–1.034)	0.056	89.653
Recessive	GG vs. AA+GA	-	-	-
Overdominant	GA vs. AA+GG	0.231 (0.061–0.867)	0.030	88.373
Additive	A	0.422 (0.137–1.303)	0.134	91.082
rs10168266				
Codominant	CT vs. CC	1.029 (0.342–3.091)	0.960	95.291
	TT vs. CC	0.600 (0.035–10.195)	0.724	
Dominant	CT+TT vs. CC	0.975 (0.339–2.806)	0.963	93.420
Recessive	TT vs. CC+CT	0.595 (0.036–9.943)	0.718	93.293
Overdominant	CT vs. CC+TT	1.051 (0.352–3.135)	0.929	93.415
Additive	C	0.930 (0.372–2.321)	0.876	93.398
Exudative AMD				
rs10181656				
Codominant	CG vs. CC	1.548 (0.544–4.410)	0.413	104.092
	GG vs. CC	0.516 (0.093–2.854)	0.448	
Dominant	CG+GG vs. CC	1.239 (0.478–3.213)	0.660	103.478
Recessive	GG vs. CG+CC	0.442 (0.083–2.359)	0.339	102.779
Overdominant	CG vs. CC+GG	1.676 (0.603–4.664)	0.322	102.661
Additive	C	0.977 (0.467–2.044)	0.952	103.669
rs7574865				
Codominant	GT vs. GG	1.500 (0.528–4.265)	0.447	103.326
	TT vs. GG	0.333 (0.051–2.200)	0.254	
Dominant	GT+TT vs. GG	1.150 (0.443–2.987)	0.774	103.590
Recessive	TT vs. GG+GT	0.289 (0.045–1.849)	0.190	101.918
Overdominant	GT vs. GG+TT	1.676 (0.603–4.664)	0.322	102.661
Additive	G	0.886 (0.416–1.888)	0.754	103.576
rs7601754				
Codominant	GA vs. AA	0.865 (0.311–2.409)	0.782	104.817
	GG vs. AA	-	-	
Dominant	GA+GG vs. AA	0.923 (0.334–2.550)	0.877	103.649
Recessive	GG vs. AA+GA	-	-	-
Overdominant	GA vs. AA+GG	0.844 (0.303–2.346)	0.745	103.568
Additive	A	1.006 (0.386–2.618)	0.990	103.673
rs10168266				
Codominant	CT vs. CC	0.673 (0.225–2.017)	0.480	105.180
	TT vs. CC	0.857 (0.073–10.064)	0.902	
Dominant	CT+TT vs. CC	0.696 (0.246–1.969)	0.495	103.214
Recessive	TT vs. CC+CT	0.943 (0.082–10.901)	0.963	103.671
Overdominant	CT vs. CC+TT	0.679 (0.228–2.018)	0.486	103.195
Additive	C	0.778 (0.331–1.824)	0.563	103.344

*p*—Significance level and Bonferroni-corrected significance level when *p* < 0.05/4; OR—odds ratio; CI—confident interval; AIC—Akaike information criteria. * 95% confidence interval (CI) of the mean is a range with an upper and lower number calculated.

**Table 14:** Serum STAT4 level associations with STAT4 SNPs.

Genotype	Serum STAT4 Levels			*p*-Value
	Early AMD Mean (Std. Deviation)	Exudative AMD Median (IQR)	Control Median (IQR)	
*STAT4* rs10181656				
CC	0.431 (-)	0.109 (0.038)	0.202 (0.560)	0.555 ^1^ 0.405 ^2^
CG+GG	0.256 (0.198)	0.178 (0.823)	0.292 (0.209)	0.826 ^1^ 0.011 ^2^
*STAT4* rs7574865				
GG	0.182 (0.198)	0.194 (0.123)	0.324 (0.318)	0.728 ^1^ 0.054 ^2^
GT+TT	0.356 (0.188)	0.198 (0.353)	0.292 (0.209)	0.756 ^1^ 0.062 ^2^
*STAT4* rs7601754				
AA	0.305 (0.187)	0.221 (0.198)	0.458 (0.268)	0.631 ^1^ 0.858 ^2^
GA+GG	0.176 (0.124)	0.174 (0.155)	0.258 (0.144)	0.972 ^1^ 0.889 ^2^
*STAT4* rs10168266				
CC	0.256 (0.266)	0.152 (0.190)	0.268 (0.268)	0.821 ^1^ 0.658 ^2^
CT+TT	0.255 (0.178)	0.185 (0.562)	0.268 (0.154)	0.956 ^1^ 0.039 ^2^

^1^ Early AMD vs. control group. ^2^ Exudative AMD vs. control group.

## Data Availability

Data will be sent upon a request.

## References

[1] García-Layana A., Cabrera-López F., García-Arumí J., Arias-Barquet L., Ruiz-Moreno J.M. (2017). Early and intermediate age-related macular degeneration: Update and clinical review. Clin. Interv. Aging.

[2] Cho Y.K., Park D.H., Jeon I.C. (2021). Medication Trends for Age-Related Macular Degeneration. Int. J. Mol. Sci..

[3] Schnabolk G. (2019). Systemic Inflammatory Disease and AMD Comorbidity. Advances in Experimental Medicine and Biology.

[4] Fan Q., Maranville J.C., Fritsche L., Sim X., Cheung C.M.G., Chen L.J., Gorski M., Yamashiro K., Ahn J., Laude A. (2017). HDL-cholesterol levels and risk of age-related macular degeneration: A multiethnic genetic study using Mendelian randomization. Int. J. Epidemiol..

[5] Zhang Q.Y., Tie L.J., Wu S.S., Lv P.L., Huang H.W., Wang W.Q., Wang H., Ma L. (2016). Overweight, Obesity, and Risk of Age-Related Macular Degeneration. Investig. Ophthalmol. Vis. Sci..

[6] Deng Y., Qiao L., Du M., Qu C., Wan L., Li J., Huang L. (2021). Age-related macular degeneration: Epidemiology, genetics, pathophysiology, diagnosis, and targeted therapy. Genes Dis..

[7] Bruneau S., Nakayama H., Woda C.B., Flynn E.A., Briscoe D.M. (2013). DEPTOR regulates vascular endothelial cell activation and proinflammatory and angiogenic responses. Blood.

[8] Qing M., Schumacher K., Heise R., Wöltje M., Vazquez-Jimenez J.F., Richter T., Arranda-Carrero M., Hess J., von Bernuth G., Seghaye M.-C. (2003). Intramyocardial synthesis of pro- and anti-inflammatory cytokines in infants with congenital cardiac defects. J. Am. Coll. Cardiol..

[9] Torpey N., Maher S.E., Bothwell A.L., Pober J.S. (2004). Interferon alpha but not interleukin 12 activates STAT4 signalling in human vascular endothelial cells. J. Biol. Chem..

[10] Liang Y., Pan H.-F., Ye D.-Q. (2014). Therapeutic potential of STAT4 in autoimmunity. Expert Opin. Ther. Targets.

[11] Darnell J.E. (2003). Signal Transducers and Activators of Transcription (STATs).

[12] Loh C.Y., Arya A., Naema A.F., Wong W.F., Sethi G., Looi C.Y. (2019). Signal transducer and activator of transcription (STATs) proteins in cancer and inflammation: Functions and therapeutic implication. Front. Oncol..

[13] Vidangos N., Maris A.E., Young A., Hong E., Pelton J.G., Batchelor J.D., Wemmer D.E. (2013). Structure, function, and tethering of DNA-binding domains in σ54 transcriptional activators. Biopolymers.

[14] Yang C., Mai H., Peng J., Zhou B., Hou J., Jiang D. (2020). STAT4: An immunoregulator contributing to diverse human diseases. Int. J. Biol. Sci..

[15] Mehrpouya-Bahrami P., Moriarty A.K., de Melo P., Keeter W.C., Alakhras N.S., Nelson A.S., Hoover M., Barrios M.S., Nadler J.L., Serezani C.H. (2021). STAT4 is expressed in neutrophils and promotes antimicrobial immunity. JCI Insight.

[16] Cui J., Tong R., Xu J., Tian Y., Pan J., Wang N., Chen H., Peng Y., Fei S., Ling W. (2021). Association between STAT4 gene polymorphism and type 2 diabetes risk in Chinese Han population. BMC Med. Genom..

[17] Cui J., Tong R., Xu J., Tian Y., Pan J., Wang N., Chen H., Peng Y., Fei S., Ling W. (2020). STAT4 sequence variant and elevated gene expression are associated with type 1 diabetes in Polish children. Cent. Eur. J. Immunol..

[18] Shi Z., Zhang Q., Chen H., Lian Z., Liu J., Feng H., Miao X., Du Q., Zhou H. (2017). *STAT4* Polymorphisms are Associated with Neuromyelitis Optica Spectrum Disorders. Neuromol. Med..

[19] Al-Zamil W.M., Yassin S.A. (2017). Recent developments in age-related macular degeneration: A review. Clin. Interv. Aging.

[20] Zheng J., Yin J., Huang R., Petersen F., Yu X. (2013). Meta-analysis reveals an association of *STAT4* polymorphisms with systemic autoimmune disorders and anti-dsDNA antibody. Hum. Immunol..

[21] Ghanavati F., Nezhad S.R.K., Hajjari M.R., Akhoond M.R. (2019). Association of Signal Transducer and Activator of Transcription 4 rs10181656 Polymorphism with Rheumatoid Arthritis and Systemic Sclerosis in Khuzestan Province in Southwestern Iran. Arch. Rheumatol..

[22] Hellquist A., Sandling J.K., Zucchelli M., Koskenmies S., Julkunen H., D'Amato M., Garnier S., Syvänen A.-C., Kere J. (2010). Variation in STAT4 is associated with systemic lupus erythematosus in a Finnish family cohort. Ann. Rheum. Dis..

[23] Beltrán Ramírez O., Mendoza Rincón J.F., Barbosa Cobos R.E., Alemán Ávila I., Ramírez Bello J. (2016). STAT4 confers risk for rheumatoid arthritis and systemic lupus erythematosus in Mexican patients. Immunol. Lett..

[24] Myrthianou E., Zervou M., Budu-Aggrey A., Eliopoulos E., Kardassis D., Boumpas D., Kougkas N., Barton A., Sidiropoulos P., Goulielmos G. (2016). Investigation of the genetic overlap between rheumatoid arthritis and psoriatic arthritis in a Greek population. Scand. J. Rheumatol..

[25] Yan N., Meng S., Zhou J., Xu J., Muhali F.S., Jiang W., Shi L., Shi X., Zhang J. (2014). Association between *STAT4* Gene Polymorphisms and Autoimmune Thyroid Diseases in a Chinese Population. Int. J. Mol. Sci..

[26] Gu E., Lu J., Xing D., Chen X., Xie H., Liang J., Li L. (2015). Rs7574865 polymorphism in signal transducers and activators of transcription 4 gene and rheumatoid arthritis: An updated meta-analysis of 28 case-control comparisons. Int. J. Rheum. Dis..

[27] Liang Y.L., Wu H., Shen X., Li P.Q., Yang X.Q., Liang L., Tian W.H., Zhang L.F., Xie X.D. (2012). Association of STAT4 rs7574865 polymorphism with autoimmune diseases: A meta-analysis. Mol. Biol. Rep..

[28] Yuan H., Feng J.-B., Pan H.-F., Qiu L.-X., Li L.-H., Zhang N., Ye D.-Q. (2014). A meta-analysis of the association of STAT4 polymorphism with systemic lupus erythematosus. Mod. Rheumatol..

[29] Chai H.C., Chua K.H., Lim S.K., Phipps M.E. (2014). Insight into gene polymorphisms involved in toll-like receptor/interferon signalling pathways for systemic lupus erythematosus in South East Asia. J. Immunol. Res..

[30] Yang C., Chen H., Zhou B., Yin J., Cao G., Hou J., Jiang D. (2022). The effects of the interactions of *STAT4* rs7574865 with HBV mutations on the risk of hepatocellular carcinoma. Mol. Carcinog..

[31] Shi H., He H., Ojha S.C., Sun C., Fu J., Yan M., Deng C., Sheng Y.J. (2019). Association of STAT3 and STAT4 polymorphisms with susceptibility to chronic hepatitis B virus infection and risk of hepatocellular carcinoma: A meta-analysis. Biosci. Rep..

[32] Gao W., Dong X., Yang Z., Mao G., Xing W. (2020). Association between rs7574865 polymorphism in *STAT4* gene and rheumatoid arthritis: An updated meta-analysis. Eur. J. Intern. Med..

[33] Wang J.M., Xu W.D., Huang A.F. (2020). Association of STAT4 Gene Rs7574865, Rs10168266 Polymorphisms and Systemic Lupus Erythematosus Susceptibility: A Meta-analysis. Immunol. Investig..

[34] Abdelmajed S.S., El-Dessouky M.A., SalahElDin D.S., Hassan N.A.M., Zaki M.E., Ismail S. (2021). Assessing the association of rs7574865 STAT4 gene variant and type 1 diabetes mellitus among Egyptian patients. J. Genet. Eng. Biotechnol..

[35] Ebrahimiyan H., Rezaei R., Mostafaei S., Aslani S., Goulielmos G.N., Jamshidi A., Mahmoudi M. (2018). Association study between STAT4 polymorphisms and susceptibility to systemic lupus erythematosus disease: A systematic review and meta-analysis. Meta Gene.

[36] Kawasaki A., Ito I., Hikami K., Ohashi J., Hayashi T., Goto D., Matsumoto I., Ito S., Tsutsumi A., Koga M. (2008). Role of *STAT4* polymorphisms in systemic lupus erythematosus in a Japanese population: A case-control association study of the STAT1-STAT4 region. Arthritis Res. Ther..

